# Supplementary material for: Are carbon emissions trading and green financial instruments synergistic? -Comprehensive quantitative research based on content analysis
Source: PLoS One. 2024 Mar 7;19(3):e0298601. doi: 10.1371/journal.pone.0298601 (PMC10919601; doi:10.1371/journal.pone.0298601)
Supplement: S1 Data — (ZIP) [file pone.0298601.s001.zip › Data/地级市Z_F工作报告.zip!/┴╔─■/╠·┴δ╩╨/└┤╫╘╣1⁄2╓┌║┼í╛┬φ┐╦╩2╛▌═°í┐_2017.docx]

各位代表：

现在，我代表市人民政府向大会报告工作，请予审议，并请市政协委员和列席同志提出意见。

一、2016年工作回顾

刚刚过去的一年，是铁岭发展形势最为严峻、承受压力最大、面临困难最多的一年。我们在省委、省政府和市委的坚强领导下，团结和带领全市各族人民，积极适应经济发展新常态，坚持稳中求进工作总基调，以实施供给侧结构性改革为主线，统筹推进稳增长、促改革、调结构、惠民生、防风险各项工作，保持了经济社会平稳发展态势，一些重点工作实现了新突破。

—— 项目年工作取得新成效连续12年开展项目年活动，我市工作经验在全省推广。开展走出去、请进来重大招商活动18次，新签约重点项目127个，到位资金83.5亿元。向上争取各类无偿资金140亿元，申请专项建设基金12.9亿元。

全市开（复）工亿元以上项目178个。中利祥晖光伏发电、豪唐纸业等重点项目进展顺利，冠林汽车零部件、耐克森斯汽车线束、春天药业等一批项目投产运营。30万吨燃料乙醇、东北杂粮交易中心项目加快推进。

建立了PPP项目储备库，在库项目89个，计划总投资322亿元。古榆树煤田煤层气、铁南全域城镇化等6个10亿元以上重大项目纳入东北振兴三年滚动计划。铁岭、四平、通辽、辽源经济协作区建设开始启动。

铁岭保税物流中心顺利通过国家验收并封关运行。我市与马来西亚巴生港自贸区签订了战略合作协议。组建了铁岭市走出去企业联盟，华威石油机械制造等4家企业实现境外投资。全市进出口总额实现3.8亿美元。

—— 产业优化升级步伐加快

供给侧结构性改革稳步推进，“三去一降一补”取得初步成效，三次产业结构进一步优化。

农业农村经济健康发展。粮食生产再获大丰收，总产量达到93亿斤。蔬菜总产量突破300万吨。榛林总面积发展到113万亩，产量3.7万吨，我市获批筹建辽宁省榛子产业知名品牌示范区。落实玉米生产者补贴资金8.9亿元、种植业结构调整补贴资金7387.6万元。农机总动力提高到280万千瓦，主要农作物耕种收综合机械化水平达到85%。防汛抗旱工作扎实开展，气象信息进村入户、气象服务和预报预警工作走在全省前列。新建高标准生态养殖场30个，辉山乳业奶牛养殖量突破2万头，嬴德肉禽出口额增长25%，畜牧业规模化、标准化水平明显提升。农业产业化水平稳步提高，农产品加工园区加快发展，入驻企业170户，全市规模以上农业产业化企业实现销售收入290亿元。玉米良种繁育及加工基地项目获批国家级农业综合开发项目。农村土地流转面积达到400万亩，国家级农民合作社示范社发展到24个，省级示范家庭农场119家。建档立卡农村贫困人口减少27294人，贫困村退出45个。西丰县成为第一批国家粮改饲试点县和全国唯一永久性鹿产品加工贸易区，创建国家级农业可持续发展示范区工作取得新进展。

工业运行质量稳中有升。全市规模以上工业企业281户，实现增加值105亿元。比科新能源、中利科技等5户企业进入省级智能制造及智能服务试点示范行列。乾丰专用车首批50辆新能源公交车下线。清河区、铁岭县光伏发电项目并网发电。三浦汽车电子、中利光电新材料等13个投资3000万元以上的工业项目竣工投产。金正大三期、陆发机械等52个投资500万元以上的技改项目启动实施。成功嫁接改造20个停产停建项目，盘活土地1143亩、厂房15.8万平方米。全年开发新产品376项，民生智能抄表系统等18项产品和技术荣获省级专精特新称号，中油机械等12个产品被评为辽宁省名牌。申请发明专利200件，组建科技公共服务平台3个、科技服务机构10个、创新创业导师团队20个。沈阳工业大学铁岭研究院挂牌成立。3个众创空间进入全省首批“云启众创”行列。专用车生产基地被确定为省军民融合特色产业基地，产品展示平台上线运营。2个省级质检中心获批筹建。铁岭石油装备、开原起重机械制造2个特色产业基地通过国家科技部火炬中心复核。

现代服务业发展势头良好。服务业集聚区七个一工程加快实施，哥伦布商业综合体、凡河生态文化旅游谷等重点项目进展顺利。东北物流城实现销售收入22.8亿元，辽宁二手车市场交易车辆4.2万台。铁岭绿源农副产品物流配送有限公司投入运营，生产基地直达零售终端的流通新模式基本形成。邮农丰经营模式在全省推广。中珍网、铁岭绿农商务港等10个电商平台平稳运营。107家农事企业产品进入淘宝、京东等网站营销，200余种农产品打入省内外55家大型超市。金融广场合作机构发展到99家，注册用户达1490户。新增新三板、区域股权交易中心挂牌企业9家。昌图县、开原市、铁岭县“两权”抵押贷款试点工作稳步推进。全市存款余额1232亿元，贷款余额843亿元，分别增长9.8%和4.2%。旅游业五个一工程扎实开展，建成高标准农家乐示范村8个，象牙山温泉度假村、龙源温泉度假区投入运营。30余个大型旅游节会活动成功举办。中国铁岭荷花文化旅游节展出莲属植物619种，创吉尼斯世界纪录。全市旅游业总收入实现132.9亿元，增长13.2%。

—— 城乡环境持续改善

《铁岭市新型城镇化规划》编制完成。城市管理体制得到理顺，凡河新区划归铁岭县管理。铁岭高铁西站广场改扩建工程竣工。光荣街、东北城大道改造等工程顺利完成。城市供水2025工程启动实施。新改建供暖、供水、燃气管网138公里，中心城区实现集中供热全覆盖。城区绿化覆盖率达到39.4%。维修改造各级公路834.2公里，新三线二期和沈四高速公路（铁岭段）改扩建工程竣工通车。

实施棚户区改造4213户，货币化安置率达到67.6%。改造弃管楼329栋、125.5万平方米。商品房销售136.4万平方米，待售面积逐步减少。改造农村危房1700户，打造美丽乡村示范村25个。

生态环境治理成效显著。31个节能减排示范项目启动实施。更新新能源公交车210辆，淘汰黄标车及老旧车辆9596台。拆除建成区10吨及以下燃煤小锅炉94台。秸秆禁烧工作成效明显，综合利用水平进一步提高。辽河干流铁岭段生态封育面积18.2万亩。全市植树造林19.2万亩，昌图县获评全国绿化模范县。银州区铁西干渠治理与水系连通工程获国家支持并开工建设。凡河水利风景区晋升为国家级水利风景区。

—— 各项改革全面深化

“放管服”改革取得新进展，取消调整行政职权176项。市行政审批服务局组建完成并开始运行，42个部门263项行政审批和其他权力事项进驻中心办理。市县乡村四级联动公共行政服务体系进一步完善，在全省率先实现乡级服务中心和村级便民服务室全覆盖。中介服务机构事中事后监管得到规范，“双随机、一公开”工作成效显著。

工商登记制度改革取得初步成效，“五证合一、一照一码”营业执照办理时限压缩至3个工作日，新增内资市场主体2.1万户。央企“三供一业”分离和市属国有企业集团组建工作稳步推进，市属经营性国有资产实现集中统一监管。营改增工作深入开展，减轻企业负担3亿元以上。

土地确权登记全面铺开，我市农村产权流转交易市场建设经验在全省推广。供销合作社综合改革、集体林权制度改革稳步实施。

公车改革基本完成。事业单位分类改革正式启动。农信社改制等其他各项改革有序开展。

—— 民生和社会事业全面发展

坚守民生底线，克服财政收支矛盾突出影响，多渠道筹措资金100亿元以上，保证了教育、社保等民生资金需求。棚户区改造、提高最低工资标准等25件民生实事得到落实。

新增城镇就业1.6万人，零就业家庭保持动态为零。新农合、城镇居民医保政府补助标准同步提高到420元。城乡低保月平均保障标准分别提高到483元和309元。农村五保户集中供养年标准提高到6592元。实施贫困残疾人家庭无障碍改造850户。城镇居民人均可支配收入实现21800元，增长5.4%。农村居民人均可支配收入12400元，增长6.2%。

全市连锁幼儿园发展到100所，普惠性学前教育覆盖率达到70%。改造农村义务教育薄弱学校校舍20万平方米。银州区和市直捆绑创建义务教育发展基本均衡县（市）区工作通过国家验收。铁岭县被评为全国中小学校责任督学挂牌督导创新县。市教育局先后被教育部授予教育事业特别贡献奖和全国教育系统关心下一代工作先进集体。辽宁工程职业学院成为世界技能大赛国家集训基地。

基层医疗卫生服务体系不断健全，县级公立医院改革进一步深化。全面二孩政策有效落实。疾病防控和卫生计生监督水平不断提升。爱国卫生运动深入开展，铁岭市和西丰县分别通过省级、国家级卫生城市验收。

“辽疆情·中国梦”文化援疆演出、百幅大型工笔画创作、铁岭欢歌等活动圆满完成，“荷韵墨香”两岸书画节成功举办，11个小品小戏在央视播出。《胡萝卜村官》《寻找》《那片柞树林》等作品在国际微电影节获奖。颁金节、秋夕节等各具特色的民族文化活动蓬勃开展。全国首家农村雷锋纪念馆建成开馆。第三届中国北方体育舞蹈公开赛和首届全民广场健身舞大赛成功举办。我市运动员赵慧馨获2016年射击世界杯女子步枪第三名。

精神文明创建活动深入推进，孝道文化活动扎实开展，我市连续三次获全国双拥模范城称号。

—— 政府自身建设得到加强

坚持每季度向市委报告工作。自觉接受市人大及其常委会的法律监督、工作监督和市政协的民主监督。全年办理人大议案、人大代表建议和政协提案531件，见面率、办复率和满意率达到99%以上，落实率进一步提升。认真听取各民主党派、工商联和无党派人士意见，一些意见和建议纳入市政府决策。

权责清单、财政预决算等九大领域的政府信息公开工作扎实推进。全面推行差异化考核，绩效管理合理性不断提高。政务督查力度持续加大，各项重点工作有效落实。

“两学一做”学习教育深入开展。中央巡视回头看和省委巡视反馈意见逐一整改。全面从严治党主体责任和党风廉政建设一岗双责有效落实。严格贯彻执行中央八项规定精神，修订完善了《市政府党组工作规则》等一系列规章制度，各项工作和决策程序进一步规范。

基层组织建设进一步夯实。新改扩建基层组织活动场所413个。1175个行政村村民委员会换届选举工作圆满完成。铁岭县成为全省首批农村社区建设实验县。

信访积案化解工作积极推进，民心网诉求办理工作全省排名第一。法治宣传广泛开展，依法治市工作积极推进。组建企事业单位应急组织3285个，应急保障能力得到加强。安全生产工作扎实有效，总体形势保持平稳。食品药品安全监管体系逐步完善。社会治安防控体系不断健全，人民群众安全感和幸福感明显提升。

民族、宗教、审计、外事、侨务、综合执法、人防、物价、地震、国家安全、档案、烟草、检验检疫、武警、消防、通信、供电、驻外联络、市志、仲裁、老龄等工作也都取得了新成绩。

过去一年的成绩来之不易，这是省委、省政府和市委正确领导的结果，是市人大及其常委会法律监督、工作监督和市政协民主监督与大力支持的结果，更是全市广大干部群众齐心协力、共同奋斗的结果。在此，我代表市人民政府，向辛勤工作在各条战线上的全市各族人民，向积极支持政府工作的市人大代表、市政协委员、各民主党派、工商联、无党派人士、各人民团体、中省直驻铁单位和各界人士，向解放军驻铁部队指战员和武警官兵，向所有关心、支持、参与铁岭振兴发展的海内外朋友，表示衷心的感谢并致以崇高的敬意！

在看到成绩的同时，我们也清醒地看到经济社会发展中存在的问题和挑战，特别是主要经济指标完成得不够理想，个别指标降幅较大。预计，全市地区生产总值完成560亿元，下降24.4%；固定资产投资122亿元，下降69.3%；一般公共预算收入46.4亿元，下降7.7%；社会消费品零售总额320亿元，下降21.5%。这既有新常态下供需结构性矛盾带来的深刻变化，又有我市产业层次低、创新能力弱造成的影响；既有落实宏观调控和改革举措形成的政策性减收，又有我们坚持实事求是、挤水做实指标数据的主动作为。深入分析面临的矛盾和问题，主要表现在：一是产业结构、经济结构不适应市场变化，实体经济发展困难较多，以传统产业为代表的旧动能推动作用逐渐减弱，拉动经济增长的新动能还没有形成规模力量。二是受政策、环境和工作力度等诸多因素制约，招商引资难度加大，项目储备严重不足，新开工项目数量不多、投资额度不大，支撑铁岭未来发展的基础比较薄弱。三是财政收支矛盾突出，政府债务负担沉重，潜在的社会风险和历史遗留问题比较突出，保工资、保运转、保民生压力巨大。四是政府自身建设还需加强，特别是面对新常态、新要求，一些同志在思想上、行动上还没有及时调整过来，不相适应、不在状态、不敢担当等问题比较突出。此外，政务环境和营商环境还有待进一步改善。这些问题已经引起我们高度重视，并将在今后工作中认真加以解决。

二、2017年工作安排

今年是实施“十三五”规划、全面建成小康社会的关键之年，也是我们振奋精神、加快铁岭振兴发展的关键之年。中央连续出台一系列东北振兴政策文件，为我们加快发展提供了难得的历史机遇；省委、省政府以求真务实、敢于担当的精神，全面做实各项数据指标，经济发展步入健康轨道；市第七次党代会和市委七届二次全会胜利召开，为今后发展明确了任务、指明了方向；特别是求发展、盼振兴、尽快摆脱当前发展困境，已经成为全市三百万人民的共同期盼。只要我们顺应民意，集中民智，抢抓机遇，乘势而上，就一定能够开创铁岭振兴发展的新局面！

全市经济社会发展的总体要求是：以党的十八大和十八届六中全会、中央经济工作会议精神为指导，深入学习贯彻习近平总书记系列重要讲话精神，全面落实省十二次党代会和全省经济工作会议部署，按照“四个全面”战略布局要求，牢固树立“五大发展理念”，坚持稳中求进工作总基调，积极适应把握引领新常态，以“四个着力”为遵循，以提升经济发展质量和效益为目标，继续推进供给侧结构性改革，着力振兴实体经济，坚定不移扩大开放，持续保障和改善民生，为全面建成小康社会而努力奋斗。

主要预期目标是：全市地区生产总值增长6.5%，固定资产投资增长12%，一般公共预算收入增长5%，规模以上工业增加值增长7%，社会消费品零售总额增长8%，城镇居民人均可支配收入增长6.5%，农村居民人均可支配收入增长7%。重点抓好以下6项工作：

（一）抓开放上项目，进一步夯实经济增长基础

抢抓国家新一轮东北振兴重大机遇，积极融入国家战略，以构建大开放格局为目标，以项目年活动为载体，继续加大项目包装、引进、建设和向上争取资金工作力度，实现投资的有效增长，以项目补短板、增后劲。

认真落实中央7号和国务院28号、62号等文件精神，围绕基础设施建设、区域经济合作、产业转型升级等策划项目，重点包装储备调兵山采煤沉陷区治理、“铁四通辽”经济协作区建设等各类项目100个以上。

深耕沈吉黑、京津冀、长三角、珠三角等重点地区，进一步创新招商方式，提高招商质量和效益。组织大型招商活动20次以上，积极推动甲阳集团金膜城、马迭尔冰雪文化产业园等项目签约落地。全年到位内资110亿元，实际利用外资2亿美元，争取各类无偿资金100亿元以上。

积极推进30万吨燃料乙醇、东北杂粮交易中心等项目开工建设，力争辉山乳业、茅台保健酒、铁路物流基地等项目竣工投产。继续抓好银州区铁西老工业区和调兵山晓明独立工矿区改造搬迁项目建设，积极推进铁岭全域城区老工业区改造搬迁。

抓好铁岭保税物流中心运营和管理，全力打造对外开放新平台。支持现有出口企业做大做强，着力培育和引进一批外向型企业，进一步扩大进出口总量。全市外贸出口增幅力争达到全省平均水平。

（二）转方式调结构，深入推进供给侧结构性改革

坚持“加减乘除”一起做，深入实施“三去一降一补”，支持实体经济发展，进一步夯实产业基础，实现有效益、有质量、可持续增长。

大力发展现代农业。稳步推进农业供给侧结构性改革，以增加绿色优质农产品供给为目标，以农产品标准化生产、品牌创建和质量安全监管为抓手，以沈铁现代农业示范带和国家级农业科技示范园区建设为先导，加快转变农业发展方式，逐步提升有效供给和综合效益，推进农业大市向农业强市转变。继续巩固粮食产能。开展粮食生产全程机械化行动，完善气象防灾减灾体系，再夺农业丰产丰收。加大种植业结构调整力度，继续调减玉米种植面积。依托大型企业和规模农业经营主体，重点培育“铁稻”等农产品品牌。建设生态畜牧强市。支持国美、嬴德等骨干企业做大做强，努力将昌图县、西丰县、开原市建成全省乃至全国闻名的生猪、奶源大县和肉鸡出口基地。加快发展特色产业。积极扶持设施蔬菜、苗木花卉、寒富苹果、胡萝卜、梅花鹿、柞蚕、中草药等产业发展，争创全国榛子知名品牌示范区。提升农业产业化水平。以农产品加工园区建设为载体，积极引进和培育农产品精深加工项目，力争规模以上农业产业化企业发展到160家，销售收入突破300亿元。大力培育新型农业经营主体。支持科技型农业合作社发展，规范土地经营权流转，推进多种形式的适度规模经营。

深入实施工业强市战略。进一步强化工业主导地位，坚持以沈铁工业走廊为载体，以现有工业园区和产业集群为依托，做强存量，扩大增量，着力打造新能源、特色装备制造业和农产品精深加工三大基地，加快实现主导产业优化升级。深入实施两化融合示范工程。继续实施《中国制造2025铁岭行动纲要》，抓好20个智能制造及智能服务支撑项目，培育省级示范企业2户。加快工业互联网服务平台建设，推动工业向服务型制造转变。实施骨干企业培育工程。抓好重点技术改造项目50个，新增规模以上工业企业20户。积极培育名牌、名品和名企业家。实施工业园区提升工程。以完善功能、优化服务为目标，进一步加强园区基础和配套设施建设。加快专用车及汽车零部件、换热设备、通信材料等产业集群发展，力争每个产业集群都引进重点配套企业，全市新上产业链项目10个以上。实施资产存量盘活工程。继续开展重点工业园区嫁接改造盘活闲置资产三年滚动计划，完成嫁接改造项目20个，盘活土地1000亩以上。

加快现代服务业发展。以提升档次、培育特色、完善体系为目标，实施“互联网+流通”行动计划，推进电子商务与三次产业融合发展，培育消费品牌，打造消费热点。大力发展电子商务。突出抓好开原东北网贸港、西丰圆上圆等项目建设，加快特色淘宝村发展，培育市县乡村四级农产品直销运营网络体系和肉类蔬菜流通追溯体系。提升金融业发展水平。扩大金融广场服务内容和范围，力争3户企业挂牌上市。加快推动新天公司扩股重组，实现“第三方支付”牌照升级。进一步完善铁岭银行法人治理结构，积极引进战略投资者，不断壮大资产规模，早日实现跨区域经营目标。加大金融和非金融机构引进力度，力争平安养老保险、中国人寿财产保险在铁岭设立分支机构。加快新兴服务业发展。积极扶持信息、物流、养老、健康、社区家庭服务等新兴产业，启动建设铁岭市综合性老年养护中心。抓好服务业集聚区建设。继续推进哥伦布商业综合体、赢信财富广场等大型载体建设，不断完善集聚区功能，实现从规模扩张向质量提升转变。推动房地产健康平稳发展。适度开发商业地产项目，大力发展楼宇经济，努力化解存量地产。发展壮大旅游业。以组建铁岭旅游集团为牵动，推进旅游业体制机制创新。完善莲花湿地、辽河源湿地、龙源温泉度假区等大项目功能，力争凡河新区列入国家级生态湿地度假区。大力发展乡村旅游，打造铁岭特色旅游品牌。

（三）强基础重管理，进一步优化城乡环境

按照多规合一、以人为本、突出特色、产城融合、建管并重的原则，深入实施《铁岭市新型城镇化规划》，加快构建中心城区、县城及县域新城、中心镇特色镇、农村新型社区“四位一体”的城镇化发展新格局，进一步提高全市城镇化水平。

加大基础设施建设力度。对银州区主要街路和污水管网、雨污分流系统进行维修改造，加固维护柴河大桥。积极推进凡河新区泽沐商业城建设，修复如意湖、天水河驳岸。继续完善开原滨水、昌图滨湖等县域新城基础设施和产业基础，完成铁岭县铁南全域城镇化路网一期工程，增强城镇综合承载力和对农村转移人口的吸引力。积极推进沈铁城际铁路和沈康高速调兵山连接线建设。科学规划电力基础设施布局，逐步提升电力保障和输出能力。

切实加强城市管理。以提升管理效能为目标，进一步提高城市管理网格化、信息化、精细化水平。健全完善住宅小区综合管理体制机制，提高社会公众对物业管理服务的满意度。全面提升道路交通安全监控力度和智能化管理水平，不断改善城市交通环境。

加快推进生态环境建设。大力实施蓝天、碧水、青山、沃土四大工程。逐步取缔工业园区及产业聚集区分散燃煤锅炉，完成清河电厂、铁岭电厂超低排放改造，进一步推进热电联产项目。加强亮子河、马仲河流域综合整治及沿岸畜禽养殖粪污治理，辽河干流铁岭段河滨带植被覆盖率提高到90%以上，岸线自然保有率达到100%。全市完成植树造林13.8万亩，打造省级绿化示范村100个。积极推动垃圾分类管理，加快中电环保垃圾发电项目建设，加强城镇垃圾填埋场运营管理，逐步改善土壤环境质量。

（四）激活力添动力，积极稳妥推进改革创新

以构建开放包容、互利合作、重信守诺、尊商护商的营商环境为目标，以软环境建设为突破口，深入推进各项改革，大力支持创新创业，让发展动力竞相释放，让市场活力充分涌流。

深化“放管服”改革。继续精简市本级行政审批事项。抓好行政审批服务局的运行管理，稳步推进行政审批“两集中、两到位”。继续实施“双随机、一公开”监管改革，实现政府部门全覆盖。建立行政审批事项分类管理制度，推动行政许可标准化建设。完善以全程代办为手段的四级联动机制，形成上下一体、高效有序的公共行政服务体系，以良好的政务环境和营商环境激发各类市场主体活力，促进民营经济发展。

推进国资国企改革。进一步完善国有资产管理体制，加大国有资产集中统一监管力度，确保国有资产保值增值。继续抓好国有企业集团组建工作，大力发展混合所有制经济。用改革的方式妥善解决国企遗留问题。

深化农村综合改革。建立市县两级农村综合产权流转交易市场，完善农村信用体系，抓好农村“两权”抵押贷款试点，全面推进土地确权登记颁证工作。加快供销合作社综合改革。加大农信社改制力度，积极推进农商行组建工作。

统筹抓好事业单位分类、财税体制、投融资体制、风险管控机制等其他各项改革任务。

大力推进创新驱动发展。加强与省内外高校、科研院所的产学研合作，签订合作协议60项以上，提高科研成果转化应用水平和规模。推进企业知识产权战略，申请发明专利240件以上。加快中科院沈阳国家技术转移中心铁岭中心建设，支持院士工作站、工程技术研究中心发展，加大高新技术企业培育力度。组织开展“优秀专家铁岭行”活动，积极引进高端人才和海外研发团队，逐步建立高端人才服务经济发展的长效机制。

（五）惠民生增福祉，大力发展各项社会事业

坚持把增进人民福祉、扩大百姓获得感、创造和谐稳定社会环境作为政府一切工作的出发点和落脚点。越是经济形势严峻，越要高度关注民生，即使财政再困难、支出压力再大，也要确保民生投入不减。

着力办好15件民生实事。1.继续实施脱贫攻坚，建档立卡农村贫困人口减少28537人，贫困村退出63个。2.大力扶持创业就业，新增就业1.4万人。3.新农合政府补助标准人均提高40元，全面实施新农合和大病保险一站式报销服务。4.城乡最低生活保障标准分别提高6%和9%以上。5.为断保困难人员缴纳养老保险办理贴息贷款。6.举办全民读书节等文化活动，建设笼式足球场7个。7.实施棚户区改造1253户。8.维修改造弃管楼161栋。9.维修改造全市城区道路43条。10.改造中心城区污水管网35公里，新建雨污泵站6座。11.建设美丽乡村示范村30个。12.维修改造农村道路650公里。13.建设铁岭师专附属小学和60所连锁幼儿园。14.实施凡河水系湿地恢复工程。15.实施凡河新区水系治理工程。

加强就业和社会保障工作。积极推动就业创业，继续抓好高校毕业生、退役士兵、失业人员等重点群体就业工作。进一步强化社会保险基金征缴，确保各项社保待遇按时足额发放。有序推进城乡居民基本医保整合。加大对企业困难职工、低收入人群和困难家庭的帮扶工作。完善残疾人社会保障和服务体系，大力发展慈善事业，兜住民生底线。

坚持教育优先发展战略。争取铁岭县通过国家义务教育发展基本均衡县验收，开原市和西丰县通过省级验收。改善57所贫困地区义务教育薄弱学校基本办学条件。做好农民工随迁子女和农村留守儿童就学工作。继续开展“送教下乡”活动，建设省名师工作室2个以上。大力发展现代职业教育，为企业提供人才和用工保障。

加快发展卫生事业。继续完善公立医院管理体制，逐步实现新农合异地就医即时结报。开展改善医疗服务行动，不断提升医疗质量和服务水平。积极创新基层医疗服务模式，城市社区家庭医生签约服务重点人群达到60%以上。全面开展食品安全城市创建活动，保障全市人民饮食用药安全。

完善公共文化服务体系。抓好基层文化服务中心和文化场馆标准化建设，建立健全政府购买公共文化服务机制。支持铁岭文化演艺集团、铁岭文化产业集团做大做强，推进书法、绘画、剪纸等关联产业发展，促进文化与旅游深度融合，吸引和扩大文化消费。大力开展铁岭欢歌、送戏下乡等文化活动，搞好秧歌剧、小品小戏等传统艺术创作。加强文物保护工作。深入实施百万农民健身工程，办好第十一届万人健步走活动，加快推动体育产业发展。

维护社会和谐稳定。深入开展“七五”普法，坚持谁执法谁普法，进一步提升全民法治观念。积极做好信访工作，下大气力逐步解决历史遗留问题。建立健全市长热线工作制度，提高民意诉求办理质量。进一步提升应急管理科学化水平，增强突发事件应对能力。深入推进安全生产网格化和信息化管理体系建设，维护好安全生产稳定形势。大力构建全方位社会治安防控体系，确保人民群众安居乐业。

继续开展文明城市、文明单位、文明村镇、文明校园和文明家庭创建活动，着力抓好“家文化”宣传教育，注重家庭、注重家教、注重家风，推动形成社会主义家庭文明新风尚。厚植优秀传统文化根基，全面提高群众文明素质和城乡文明程度。

（六）转作风抓落实，不断开创政府工作新局面

重任千钧惟担当。应对重大挑战，战胜重大困难，完成重大使命，需要我们始终保持昂扬向上的斗志，始终保持战胜困难的勇气，始终保持迎难而上的决心，坚定不移地把铁岭振兴发展的使命扛在肩上，把万家忧乐放在心头，切实加强政府自身建设，不断提高执政能力和水平。

坚持依法行政，把政府活动全面纳入法治轨道。牢固树立法治意识，坚持法定职责必须为、法无授权不可为。自觉接受市人大及其常委会的法律监督、工作监督和市政协的民主监督，办好人大议案、人大代表建议和政协提案。认真听取各民主党派、工商联、无党派人士、各人民团体和各界人士的意见建议，广泛接受社会各方面的监督，真正让政府权力在阳光下运行。

坚持廉洁从政，深入推进反腐倡廉建设。严格执行党规党纪，驰而不息纠正四风。切实发挥市政府党组表率作用，带动全市政府系统广大党员干部讲修养、讲道德、讲诚信、讲廉耻。大力弘扬艰苦朴素的优良传统，坚持勤俭办一切事业。切实履行全面从严治党主体责任和党风廉政建设一岗双责，严肃查处违纪违法案件，树立政府良好形象。

坚持务实履职，提升政府服务水平。牢固树立功成不必在我的理念，不折腾、不做虚功，多做强基础、利长远的事。更加注重企业效益、民生效益、生态效益，把思维方式、工作方法、政策措施转到提高工作质量和效益上来，千方百计提供更加优质、便捷、均等的公共服务，全力以赴实现好、维护好、发展好最广大人民群众的根本利益。

坚持勤勉敬业，提高政府执行力和公信力。健全督查问责机制，坚决整肃庸政懒政怠政行为。健全激励机制和容错纠错机制，旗帜鲜明地为敢于担当的干部担当，为敢于负责的干部负责，让广大干部愿干事、敢干事、干成事。大力倡导恪尽职守、夙夜在公、主动作为、善作善成的工作作风，敢于直面问题，敢于动真碰硬，敢于担当负责，一步一个脚印地抓好各项工作的落实，让党中央国务院、省委省政府和市委的决策部署在铁岭大地落地生根、开花结果。

各位代表，实现铁岭加快振兴发展、全面建成小康社会的重任，历史地落在了我们肩上。我们一定要坚定信心不动摇，咬定目标不放松，在推进铁岭现代化建设的伟大事业中披荆斩棘，奋勇前行。让我们更加紧密地团结在以习近平同志为核心的党中央周围，在中共铁岭市委的坚强领导下，万众一心，振奋精神，埋头苦干，以优异成绩迎接党的十九大胜利召开！
